# Vulnerabilidad y prevención de las intoxicaciones en el adulto mayor

**DOI:** 10.15446/rsap.V25n4.109435

**Published:** 2023-07-01

**Authors:** Berta Schulz-Bañares, Nayibe Cortés-Rodríguez, Claudio Müller-Ramírez

**Affiliations:** 1 BS: QF. Ph. D. Ciencias Naturales. Departamento de Farmacia, Facultad de Farmacia, Universidad de Concepción. Concepción, Chile. bschulz@udec.cl Universidad de Concepción Departamento de Farmacia Facultad de Farmacia Universidad de Concepción Concepción Chile bschulz@udec.cl; 2 NC: MD. Esp. Toxicología Clínica. Centro de Investigación Información y Asesoría en Toxicología Clínica de Boyacá (CitoxBoy). Tunja, Colombia. citoxboy@gmail.com Toxicología Clínica Centro de Investigación Información y Asesoría en Toxicología Clínica de Boyacá Tunja Colombia; 3 CM: QF. M. Sc. Ciencias Farmacéuticas. Ph. D. Toxicología. Departamento de Farmacia, Facultad de Farmacia, Universidad de Concepción. Concepción, Chile. claudiomuller@udec.cl Universidad de Concepción Departamento de Farmacia Facultad de Farmacia Universidad de Concepción Concepción Chile claudiomuller@udec.cl

**Keywords:** Adulto mayor, poblaciones vulnerables, intoxicación, prevención primaria *(fuente: DeCS, BIREME)*, Aged, vulnerable population, poisoning, primary prevention *(source: MeSH, NLM)*

## Abstract

**Objetivo:**

Los objetivos de este trabajo son: enumerar algunos factores de vulnerabilidad del adulto mayor, identificar las intoxicaciones por diferentes sustancias químicas reportadas en este grupo etario, con énfasis en Iberoamérica, y describir estrategias de prevención de accidentes e intoxicaciones en el adulto mayor.

**Materiales y Métodos:**

Se realizó una revisión temática a partir de búsquedas en las bases de datos ScienceDirect, Scopus, Embase y BIREME OPS-OMS de los siguientes términos: "Older adults/adulto mayor", "elderly/tercera edad", "poisoning/intoxicación" y "overdoses/sobredosis". La búsqueda se restringió a artículos publicados desde el año 2000 y que incluyeran a mayores de 60 años.

**Resultados:**

Se detectó la necesidad de fortalecer un modelo de envejecimiento positivo, incluyendo estrategias de manejo social del riesgo, que prevengan, mitiguen y permitan superar las consecuencias de las inequidades identificadas. Dentro de los riesgos a los que se exponen las personas mayores están las intoxicaciones involuntarias o voluntarias, en las cuales los medicamentos están involucrados en mayor proporción. Se identificó el enfoque mayoritario en prevención de intoxicaciones en niños como población vulnerable, incluyendo en ella a los adultos mayores, pero no siendo exclusivas para este último grupo.

**Conclusiones:**

El constante crecimiento de la población adulta mayor pone de manifiesto la necesidad de contar con estrategias para la prevención de intoxicaciones en el hogar que estén dirigidas especialmente a este grupo.

## Abstract (en)

**Objective:**

The objectives of this work are to mention older adults' vulnerability factors, to identify main causes of poisoning among ibero-american older adults, and to describe accident and poisoning prevention strategies in the elderly.

**Materials and Methods:**

A theme-based review was carried out. Science Direct , Scopus , Embase, BIREME OPS - OMS data bases were used to search the following words: "Older adults/adulto mayor", "elderly/tercera edad", "poisoning/intoxicación", "overdoses/sobredosis". The search was restricted to published articles from 2000 and cases related to poisonings among older adults (> 60 years old).

**Results:**

There is a need to strengthen a positive ageing model including strategies that handle social risks associated with the identified inequities among older adults. One of the risks that face older adults is intentional or unintentional poisonings related to drugs. Most poisoning prevention strategies focus on children as vulnerable populations in which older adults are included.

**Conclusions:**

Older adult populations are growing at a constant rate therefore poisoning prevention strategies specially designed for this group are needed.

El envejecimiento de la población mundial es considerado un factor de riesgo para ser vulnerable. Para hablar de grupos vulnerables empezaremos por definir la etimología de vulnerabilidad, que tiene su origen en el latín vulnus, que significa 'herida'; la partícula -abilis, que significa 'poder de'; y el sufijo -dad, que significa 'cualidad'. De este modo, la vulnerabilidad puede ser entendida como la "cualidad que tiene alguien o algo para poder ser herido o dañado" 1.

Por otro lado, la vulnerabilidad, desde el punto de vista de atención en salud, se entiende cuando hay grupos de personas en desventaja por riqueza, poder o prestigio o determinantes sociales que se comportan como factores de riesgo que pueden afectar su salud. Algunos grupos considerados vulnerables son: adultos mayores, expresidiarios, inmigrantes, entre otros. La Organización Mundial de la Salud (OMS) entiende que los determinantes sociales en salud (DSS) son "Las condiciones en que las personas nacen, viven, trabajan y envejecen" 2. Los adultos mayores, según la OMS, son aquellas personas con una edad mayor o igual a 60 años; este grupo representa un porcentaje de la población cada más elevado en diferentes países del mundo 3.

La Organización de las Naciones Unidas (ONU), dentro de los desafíos globales, informa que la población mundial está envejeciendo, debido al aumento de la esperanza al nacer, la disminución de los niveles de fecundidad y el reporte de algunos países que presentan disminución en el número de habitantes. Estos cambios influyen en el logro de los Objetivos de Desarrollo Soste-nible (ODS) acordados a escala mundial en busca de la prosperidad económica y la prosperidad social. El 17 de junio del 2019, en un comunicado de prensa, la Organización informó que la esperanza de vida en los países menos desarrollados se situaba 7,9 años por debajo del promedio mundial, por múltiples factores: mortalidad en la niñez, mortalidad materna y violencia, entre otros.

El envejecimiento es un factor de riesgo para caer o permanecer en la pobreza, con mayor impacto en los países en desarrollo. Las bases de datos o estudios sobre la pobreza, el adulto mayor y las intoxicaciones son escasas, en mayor proporción en Latinoamérica y el Caribe 4,5.

Asociado a lo anterior, el adulto mayor generalmente está expuesto a polifarmacia, considerada como el consumo de cinco medicamentos o más, o polipatología, es decir, la presencia de dos o más enfermedades crónicas sintomáticas, y por lo tanto variables fisiológicas que pueden alterar el metabolismo de las sustancias a las que se expone, las cuales pueden llevar a efectos indeseados o intoxicaciones accidentales 3.

La OMS, dentro del plan para la Década del Envejecimiento Saludable 2020-2030, prevé que en esta década el porcentaje de habitantes mayores de 60 años aumentará 34% -en la actualidad el número de personas mayores supera el de niños menores de 5 años- y que en el 2050 el número será mayor al de adolescentes o jóvenes de 15 a 30 años. Las regiones que proyectan mayor crecimiento son África, seguida de América Latina y el Caribe. Para el 2050, casi el 80 % de la población de todo el mundo vivirá en países en desarrollo. Esto pone en evidencia que el envejecimiento de la población es mucho más rápido que en el pasado. Lo anterior crea la necesidad de diseñar estrategias que permitan el cumplimiento de los Objetivos de Desarrollo Sostenible, para un envejecimiento saludable, en un entorno oportuno y seguro para el adulto mayor en las comunidades, de la mano de las entidades gubernamentales 6.

Los objetivos de este trabajo son: 1) enumerar algunos factores de vulnerabilidad del adulto mayor en el mundo e Iberoamérica; 2) identificar las intoxicaciones por diferentes sustancias químicas reportadas, con énfasis en Iberoamérica; 3) describir estrategias de prevención de accidentes e intoxicaciones en el adulto mayor.

## MATERIAL Y MÉTODOS

Se llevó a cabo una revisión temática, siguiendo las indicaciones del manual Lineamientos revisión temática, publicado por la Universidad Nacional de Colombia 7, a partir de búsquedas en las bases de datos ScienceDirect, Scopus, Embase y BIREME-OPS-OMS, tanto en inglés como en español, de los siguientes términos: "Older adults/adulto mayor", "elderly/tercera edad", "poisoning/intoxicación" y "overdoses/sobredosis".

La revisión bibliográfica se hizo en diciembre del año 2022 y se incluyeron artículos publicados de adultos mayores relacionados con intoxicaciones accidentales o voluntarias, como también actividades de prevención al respecto. Restringimos nuestra búsqueda a artículos publicados desde el año 2000 en adelante y que incluyeran a la población mayor de 60 años. Además, la búsqueda se extendió a documentos oficiales gubernamentales y de organizaciones internacionales que se relacionaran con los objetivos de la investigación.

Después de la búsqueda inicial, los artículos fueron seleccionados si el título o resumen incluían al menos uno de los siguientes criterios:

- Población mayor de 60 años
- Identificación de factores de riesgo del adulto mayor para intoxicaciones
- Estrategias de prevención en intoxicaciones en adultos mayores
- Relacionado con población iberoamericana.

Todos aquellos artículos que no cumplían con los criterios de inclusión fueron excluidos.

## RESULTADOS

### Uso de encuestas para el adulto mayor

La Encuesta multicéntrica de salud, bienestar y envejecimiento (SABE) en América Latina, considerada el mayor estudio multicéntrico de la población de adultos mayores, que fue financiada inicialmente por la Organización Mundial de la Salud/Organización Panamericana de la Salud (OMS/OPS) incluyó en el año 2001 a 10 891 personas mayores de 60 años residentes en siete ciudades grandes de la región (Bridgetown, Buenos Aires, La Habana, Ciudad de México, Montevideo, Santiago de Chile y Sao Paulo) 8,9. Se caracterizó la salud de las personas adultas mayores a nivel geográfico, sociodemográfico y epidemiológico, analizando la calidad de vida e identificando los factores de riesgo. Se realizó la selección en dos etapas, la primera por áreas geográficas de las ciudades y la segunda de forma sistemática; la edad promedio de los encuestados fue de 69,9 a 73,3 años y predominó el género femenino (58,9%). El 72,9% de los adultos mayores vivían acompañados. En Buenos Aires y Montevideo más de la tercera parte de los encuestados consideró que su salud no era buena, percepción que aumentó en el resto de las ciudades: Bridgetown (49,1%), Sao Paulo (53,4%), La Habana (62,6%), Santiago de Chile (63,2%) y Ciudad de México (69,4%). El mayor nivel educacional se asoció con menor frecuencia de discapacidad solamente en Buenos Aires y Santiago 8,10.

La encuesta SABE de Ecuador, realizada en 2009-2010, incluyó a personas mayores de 60 años, con predominio del género femenino (53,3%). El 1 % de los adultos mayores viven solos, de los cuales el 30,8% vive en malas condiciones o indigencia y el 18,8% vive con su pareja. El grado de depresión leve a moderado en mujeres fue de 40,3% y en hombres llegó al 30,3%. La prevalencia de hipertensión arterial y diabetes correspondió al 46% y al 13%, respectivamente. Según esta información, los adultos mayores ecuatorianos viven en condiciones socioeconómicas variadas 11.

En el 2022 se publicó un estudio ecológico de base secundaria, de enero de 1997 hasta diciembre del 2019, en Ecuador, que reportó las personas fallecidas de 60 años o más. Los hombres presentaron tasas de suicidio más altas en el grupo de edad de más de 80 años, mientras que las tasas de suicidio más bajas se hallaron en el grupo de 65 a 69 años. Para las mujeres, las tasas más altas se registraron en el grupo de 70 a 74 años y las más bajas en el grupo de 65 a 69 años. En el área urbana, las tasas de mortalidad en ambos géneros fueron más altas que en el área rural.

Además, un nivel educativo bajo se asoció con mayores tasas de suicidio. Esta información puede servir para establecer estrategias de prevención en el adulto mayor de acuerdo con el grupo etario objeto de intervención 12.

La encuesta SABE Colombia 2015, con una muestra representativa del país, incluyó medidas antropométricas, toma de laboratorios, un estudio cualitativo con perspectivas culturales y de género de la calidad de vida y una sub encuesta a los cuidadores. Dentro de los resultados reportados, el 57% de los adultos mayores tenían entre 60 y 69 años, seguidos con el 30% por el grupo entre 70-79 años, y con el 13 % los mayores de 80 años. La proporción de personas viudas aumenta con la edad; el 74% no se autorreconoce como perteneciente a ninguna etnia; el 30,9% trabaja; el 29,1% recibe pensión y el 20,5% subsidios estatales. Más de la mitad de la población asocia la vejez con dependencia, fragilidad y discriminación, especialmente personas solteras y de género masculino. Dentro de las conclusiones está la necesidad de protección social integral, en la que se consideren estrategias de manejo social del riesgo que prevengan, mitiguen y permitan superar las consecuencias de las inequidades identificadas 8,13.

En Colombia se llevó a cabo un estudio para identificar los factores asociados a la vulnerabilidad cognitiva de los adultos mayores en tres ciudades durante el año 2016. El riesgo de deterioro cognitivo se encontró en 5,1% de la muestra en Medellín, 2,7% en Pasto y 1,7% en Barranquilla, con predominio en hombres entre 75 y 89 años, sin pareja y con baja escolaridad. Además, el deterioro cognitivo se presentó en personas con poca actividad física, con trastornos depresivos y que requerían soporte social 14.

También se hizo un análisis trasversal analítico en el que se exploraron los factores demográficos, de salud y funcionales asociados a la depresión en el anciano, y se concluyó que la depresión está presente en una parte importante de la población de adultos mayores y que se asocia a condiciones no solo de salud sino también demográficas y de capacidad funcional 15.

En Chile, dentro del Programa de Adulto Mayor (PAM), se ha realizado varias veces la encuesta de calidad de vida: la primera en el 2007, la segunda en el 2010, la tercera en el 2013, la cuarta en el 2016 y la quinta en el 2020. Esta encuesta ha estado a cargo de la Pontificia Universidad Católica de Chile, junto al Servicio Nacional del Adulto Mayor (Senama) y la Caja Los Andes. Con el análisis de los resultados se determinaron aspectos sociales y económicos que permitieron aproximarse a la realidad de la población chilena. Dentro de las conclusiones de la última encuesta se estableció la necesidad prioritaria de fortalecer un modelo de envejecimiento positivo y cambiante con educación continua 16,17.

### Envejecimiento saludable y factores de riesgo

El envejecimiento es una realidad que impacta a todas las personas. De acuerdo con los factores de riesgo, se desarrollan comorbilidades que afectan en mayor o menor grado la calidad de vida del adulto mayor 4.

La intoxicaciones o sobredosis en personas mayores de 65 años fueron una situación clínica poco frecuente en décadas pasadas, o al menos eran percibidas de esta forma. Las intoxicaciones voluntarias o involuntarias se reportan principalmente en menores de 65 años. Sin embargo, este comportamiento ha cambiado con el tiempo.

En Chile, en las XXVII Jornadas de Salud Pública de Chile, que tuvieron lugar en el 2008, se publicaron las consultas por intoxicaciones recibidas por el Centro de Información Toxicológica de la Universidad Católica durante los años 2006 y 2007. Del total de los casos, un 4% (2 164) correspondió a personas mayores de 60 años, y dentro de este grupo, el 65% fue del sexo femenino. La principal vía de intoxicación fue la oral, la manera más frecuente fue accidental y la causa más recurrente fueron los medicamentos 18.

Por otro lado, dentro del análisis de datos de 1 384 participantes > 60 años que participaron en la Encuesta Nacional de Salud de Chile 2009-2010, se identificaron factores sociodemográficos, de estilo de vida y de salud asociados al deterioro cognitivo en adultos mayores chilenos, dentro de los que se encontró: incremento de edad, bajo nivel de escolaridad, género masculino, sedentarismo e inactividad 19.

En Costa Rica se llevó a cabo un estudio retrospectivo descriptivo que analizó el perfil epidemiológico de las consultas de intoxicaciones en personas adultas mayores, de enero del 2015 a octubre del 2020, de acuerdo con datos del Centro Nacional de Control de Intoxicaciones de Costa Rica (CNCI). Entre los resultados se observó que la vía de intoxicación más frecuente fue la oral, con un 79 % en el periodo examinado; la manera de intoxicación fue accidental y la causa principal fue el consumo de medicamentos, entre ellos benzodiacepinas, tramadol e insulina. Con respecto a la insulina, la mayoría de los eventos se relacionan con dificultades visuales o alteraciones cognitivas por parte del adulto mayor, asociado a la vía de administración y error de dosificación. La representación mayor fue de mujeres (57%). Durante el estudio se identificó un aumento de intoxicaciones por hipoclorito de sodio en los primeros nueve meses del 2020, que corresponde a la época de pandemia de covid-19 y puede estar asociado a la desinfección de superficies, donde el almacenamiento inadecuado lleva a accidentes que en el futuro pueden ser eventos prevenibles 3.

En el 2021 se publicó un estudio observacional transversal (de enero del 2011 a diciembre del 2019) de un hospital de tercer nivel en España, que consideró todas las personas atendidas por intoxicación en el servicio de urgencias. El 6,1% de los casos correspondió a pacientes mayores de 65 años, y dentro de este grupo el 88% se asoció a la presencia de un solo tóxico: alcohol (51,6%), fármacos (29,5%) o productos de uso doméstico (12,8%). Con relación a los fármacos, las benzodiacepinas representan un 11,7 % de todos los casos 20.

Entre las estrategias de prevención de estos eventos se encuentra la prescripción de medicamentos con ventana terapéutica más amplia, uso de regímenes de medicamentos simplificados, sistema de prescripción electrónica, instrucciones claras por parte del personal médico, farmacéutico o dispensador de medicamentos, y dispensadores diarios o semanales de medicamentos.

## Gestión de riesgo y estrategias de mitigación para las intoxicaciones en el adulto mayor

Al adulto mayor se le atribuyen factores de riesgo para intoxicaciones voluntarias e involuntarias, como las comorbilidades, entre las cuales se encuentra la depresión no tratada, cuya intervención adecuada puede convertirse en un factor protector ante problemas familiares, problemas con el cónyuge, polimedicación o cambios fisiológicos propios del envejecimiento que pueden alterar la farmacocinética y la farmacodinamia de los medicamentos, lo que aumenta o disminuye algunos de sus efectos, que pueden desembocar en eventos desafortunados para esta población. La población mundial está envejeciendo y este solo hecho incrementa el riesgo de intoxicaciones en esta población, lo que se asocia a una mayor probabilidad de complicaciones 3.

En Chile, durante la primera ola de la pandemia, se llevó a cabo un estudio observacional en personas mayores de 60 años, mediante encuestas y entrevistas telefónicas, que evaluó los determinantes sociales de la salud (DSS) y la resiliencia. Entre los DSS se relacionaron: edad, género, nivel educativo, condición laboral, aislamiento social, soledad, insatisfacción de necesidad de vivienda y atención en salud. La resiliencia fue definida, según la American Psychological Association (APA), como el proceso de adaptarse bien a la adversidad, a un trauma, tragedia, amenaza o fuentes de tensión significativas, como problemas familiares o de relaciones personales, problemas serios de salud o situaciones estresantes.

Se encuestó a 582 adultos mayores, con un promedio de edad de 71 años (rango 60 a 92 años), y entre los factores asociados a un bajo nivel de resiliencia se hallaron síntomas depresivos, soledad y aislamiento social. El ser mujer resultó ser un factor protector. La vulnerabilidad de este grupo etario, a escala mundial y latinoamericana, corrobora la necesidad de preparar y ejecutar estrategias específicas de educación en prevención de intoxicaciones, cuyo foco prioritario apunte al apropiado manejo y almacenamiento de los productos potencialmente tóxicos en el hogar.

La búsqueda de disponibilidad de material o información preventiva demostró que existe poca accesibilidad y disponibilidad a dicho material o información. En la Tabla 1 se resume una selección del material del tema, en la cual se observa gran variabilidad en la forma y el foco principal de la información preventiva que se entrega.

**Tabla 1:** Información de artículos de organizaciones gubernamentales disponibles sobre el tema de prevención de intoxicaciones en el adulto mayor en el hogar

Artículo (título, país, año)	Especificidad enfoque adulto mayor	Recomendaciones comunes	Aspectos destacables
Prevención envenenamientos e intoxicaciones en grupos vulnerables-México, 2017 21	Estadística de casos de intoxicación en edades "vulnerables"	Prohibir venta a granel en envases inadecuados y uso de sustancias químicas tóxicas. Promover el adecuado envasado, almacenamiento y etiquetado de productos tóxicos y medicamentos.	Se indica como "vulnerables" a población infantil, adolescentes, adultos mayores, factores de riesgo, recomendaciones generales para prevenir y reducir intoxicaciones. Descripción general de costos y papel de la salud pública. Recomendación general de primeros auxilios.
Boletín informativo: prevención de las intoxicaciones accidentales en las personas de edad avanzada eunese-ue, 2008 22	Específica para adultos mayores	Evitar intoxicaciones por medicamentos, monóxido de carbono, productos químicos domésticos.	Se entregan estadística de lesiones, lesiones por intoxicaciones accidentales, consecuencias, factores de riesgo y prevención. Se especifican consejos para adultos mayores, cuidadores, profesionales y responsables de las políticas públicas.
Prevención de intoxicaciones en niños, adultos mayores y personas con discapacidad o alteraciones cognitivas. Argentina. gob.ar, 2022 23	Población vulnerable	Evitar las intoxicaciones en el hogar: productos de limpieza, medicamentos y productos químicos.	Se refiere a población vulnerable como niños, adultos mayores y personas con trastornos cognitivos. Entrega recomendaciones para evitar las intoxicaciones con productos de limpieza, medicamentos o productos químicos.
Prevención de envenenamientos e intoxicaciones en personas mayores-España, 2020 24	Específica para personas mayores	Se dan consejos de prevención en exposición a: medicamentos (en general), gas y productos de limpieza.	Información indica que la mayoría de las intoxicaciones son por medicamentos o inhalación de gas y pueden llegar a complicaciones graves. Se dan consejos de prevención de exposición a: medicamentos, gas y productos de limpieza Breve y preciso.
Personas en riesgo: Adultos mayores Seguridad de los alimentos, Estados Unidos, 2022 25	Específica para personas mayores	Prevención de intoxicaciones por alimentos en mal estado: higiene de los alimentos.	Se explican las razones del mayor riesgo de intoxicaciones alimentarias. Da consejos de prevención, para lo cual incorpora un afiche en el que indica tres agentes patógenos comunes. Está disponible un enlace a información en español e inglés.

## DISCUSIÓN

En la realización de este trabajo surgieron las siguientes preguntas: 1) ¿qué estrategias gubernamentales existen para fomentar un envejecimiento saludable en cada uno de los países iberoamericanos?, y 2) ¿los sistemas de salud de Latinoamérica están preparados para prevenir y afrontar comorbilidades y potenciales intoxicaciones en la población adulto mayor?

Con la información recabada en este trabajo no podríamos dar respuesta a las preguntas anteriores, pero sí enumerar algunas estrategias para disminuir los riesgos en la población de adultos mayores, como se presenta a continuación:

### Uso seguro de medicamentos

1. Mantenga los medicamentos en sus envases originales.
2. Lea muy bien la información de administración del medicamento prescrito por el médico tratante (dosis, forma de administración, tiempo).
3. Administre o tome los medicamentos con buena visibilidad (encender la luz en la noche o cuando está oscureciendo), verificando el nombre del medicamento y la dosis.
4. Almacene de manera segura, fuera del alcance de niños, personas en situación de discapacidad o adultos.

### Uso seguro de pesticidas

1. Lea siempre las instrucciones antes de utilizar un producto que puede ser tóxico.
2. Lleve ropa protectora (guantes, manga larga, pantalones largos, calcetines, zapatos) si utiliza pesticidas u otras sustancias químicas.
3. Mantenga los pesticidas en su envase original y no utilice envases de alimentos para diluir o trasvasar.
4. Almacene los pesticidas en un lugar seguro, alejado de los alimentos.
5. No utilice los envases vacíos de pesticidas para almacenar otros productos químicos o agua.
6. No mezcle los diferentes pesticidas, ello puede aumentar su toxicidad.

### Uso seguro de otras sustancias químicas

1. Mantenga los productos químicos (limpieza, pinturas entre otros) en los envases o frascos originales. No almacene diferentes tipos de productos (alimentos y de limpieza) en el mismo lugar.
2. Almacene sustancias químicas fuera del alcance de los niños.
3. Evite reenvasar productos químicos o de limpieza en envases de bebidas comestibles.
4. Lea siempre las etiquetas y utilice los productos según las instrucciones del fabricante.
5. Evite mezclar productos químicos cuya combinación pueda generar sustancias de mayor toxicidad; por ejemplo: amoníaco o lejía.
6. Asegure una ventilación adecuada mientras use calentadores que utilicen gas como combustible.
7. Lleve ropa protectora (guantes, manga larga, pantalones largos, calcetines, zapatos) si utiliza pesticidas u otras sustancias químicas.

### Consumo seguro de alimentos

1. Lávese las manos y limpie los utensilios de cocina y las superficies con frecuencia, o al tocar alimentos como carne cruda, mariscos o huevos.
2. Consuma alimentos pasteurizados y verifique su fecha de vencimiento.
3. Almacene los alimentos perecederos en un lugar fresco, sin humedad.
4. Separe los alimentos crudos, principalmente carnes, huevos, verduras y frutas; realice una cocción adecuada de cada alimento y refrigere de forma rápida en caso de ser necesario.
5. Utilice preferiblemente tablas diferentes para alimentos como verduras o carnes, o lávelas muy bien luego de utilizarlas con algún alimento.
6. Descongele o marine los alimentos dentro del refrigerador.

El envejecimiento de la población es considerado un factor de riesgo para ser vulnerable, situación que es abordada en siete de los estudios referenciados, sin embargo, no se encontraron políticas específicas de los entes gubernamentales sobre prevención de intoxicaciones voluntarias e involuntarias en adultos mayores.

### Limitaciones

Dentro de las limitaciones de este trabajo, solo se encontraron estudios descriptivos y no se encontraron revisiones sistemáticas o metaanálisis u otros diseños que permitieran inferir conclusiones de acuerdo con la evidencia ♣
